# The Public and Patient Engagement Evaluation Tool: forward-backwards translation and cultural adaption to Norwegian

**DOI:** 10.1186/s12891-022-05514-3

**Published:** 2022-06-09

**Authors:** Andrew Garratt, Joachim Sagen, Elin Børøsund, Cecilie Varsi, Ingvild Kjeken, Hanne Dagfinrud, Rikke Helene Moe

**Affiliations:** 1 Division for Health Services, Norwegian Institute of Public Health, Oslo, Norway; 2 Oslo Metropolitan University, P.O. Box 4, St. Olavs plass, NO-0130 Oslo, Norway; 3 Norwegian National Advisory Unit on Rehabilitation in Rheumatology, Diakonhjemmet Hospital, Oslo, Norway; 4 Department of Digital Health Research, Division of Medicine, Oslo University Hospital, Oslo, Norway; 5 Faculty of Health and Social Sciences, University of South-Eastern Norway, Drammen, Norway

**Keywords:** Outcome measure, Patient engagement, Health care, Translation, Rehabilitation

## Abstract

**Background:**

Patient engagement is recommended for improving health care services, and to evaluate its organisation and impact appropriate, and rigorously evaluated outcome measures are needed.

**Methods:**

Interviews (*N* = 12) were conducted to assess relevance of the Canadian Public and Patient Engagement Evaluation Tool (PPEET) in a Norwegian setting were performed. The tool was translated, back translated, and assessed following cognitive interviews (*N* = 13), according to the COSMIN checklist. Data quality was assessed in a cross-sectional survey of patient advisory board members from different rehabilitation institutions (*N* = 47).

**Results:**

Interviews with patient board representatives confirmed the relevance of the PPEET Organisational questionnaire in a Norwegian setting and contributed five additional items. Translation and back translation of the original PPEET showed no major content differences. Differences in vocabulary and sentence structure were solved by discussion among the translators. Comments from cognitive interviews mainly related to the use of different synonyms, layout, and minor differences in semantic structure. Results of the cross-sectional survey support the data quality and construct validity of PPEET items, including 95 score comparisons where 76 (80%) were as hypothesized.

**Conclusions:**

The PPEET Organisational questionnaire has been thoroughly translated and tested, and the resulting *Evalueringsverktøy for Brukermedvirkning* (EBNOR) has adequate levels of comprehensibility and content validity. Further testing for measurement properties is recommended, but given these results, the EBNOR should be considered for assessing patient engagement in a Norwegian health care organisational context.

## Background

Patient engagement has been advocated internationally over the past decades [1], and refers to active participation to strengthen the health care services [2, 3]. However, few studies have formally evaluated the organisation and compared the impact of these initiatives [4]. Potential barriers to such evaluations include the heterogenity of the different health care systems and lack of appropriate outcome measures.

The emergence of national and international initiatives in the field of patient engagement has created a need for the development and translation of outcome measures to assess their effectiveness. There is a lack of evidence for patient engagement activities including the role of Patient Advisory Boards (PAB) in the field of healthcare. Such evidence can improve understanding of how patients experience the engagement process, whether patient engagement leads to improved quality of care, and inform practice [2].

Considerable variation in the organisation of patient engagement has been identified in Norway [5]. Interviews with PAB leaders and health care managers indicate that the impact of PABs may be higher at the regional level than in clinical practice. The impact of patient engagement is often rated higher among health care leaders than among patient representatives themselves [4, 6]. This highlights the need for more knowledge about patient engagement in the development of health care, and the need for appropriate and feasible outcome measures [4] to facilitate comparison across cultures and health care systems, as well as promote international collaboration.

Several instruments are available to assess patient and public engagement [7], but are not available in the Norwegian language. Following a review of instrument content and discussions within a research group that included patient representatives, the Public and Patient Engagement Evaluation Tool (PPEET), developed at McMaster University in Canada [8, 9], was considered as having the greatest relevance in terms of the concepts and questions it includes, for a Norwegian health care organizational setting. Moreover, PPEET development involved multidisciplinary collaboration with several stakeholders, including patient representatives, and followed a comprehensive literature review. The PPEET includes four complementary instruments with different purposes; one-time or long-term engagement activities, engagement in projects, and organisational engagement [10]. Testing for applicability and feasibility was conducted within several groups of relevant stakeholders [9].

The PPEET Organisation questionnaire is designed to assess patient representatives’ engagement and influence on decision-making processes within health care institutions. The questionnaire is generic, and hence, can be used across different health care organisations [8]. It comprises 30 items relating to different organisational domains and is divided into five parts: first, *Policies and practice*s that support engagement: second, *Participatory culture*, third, *Influence and impact*, fourth, *Collaboration and common purpose*, and fifth, *Final thoughts*. The response options include three- to five-point rating scales, in addition to free text fields. The original English PPEET has been translated into French, German and Italian [10].

The aim of this study was to translate the PPEET Organisation questionnaire from English to Norwegian and assess data quality in a survey of PAB representatives. The translation approach followed international recommendations, including qualitative pretesting of questionnaires through cognitive interviews, to assess comprehensibility, relevance and comprehensiveness of the translated items [11].

## Methods

This study included four phases: first, translation of the original PPEET questionnaire from English to Norwegian; second, interviews with PAB representatives about relevant concepts to measure from their point of view, and mapping these concepts to the PPEET; third, evaluation of PPEET translation through cognitive interviews until saturation was reached; and fourth, a cross sectional survey to assess data quality [12–15]. All data was collected from PAB representatives in Norway. The methods are summarised in Table 1. To ensure inclusion of patient representatives, interdisciplinary perspectives and current knowledge about health care, the project group modified the translated text between phases.

**Table 1:** Norwegian Public and Patient Engagement Evaluation Tool (PPEET) translation

Stepwise recommendations^a^	PPEET translation
Original version, source and translated language	English (Canada) to Norwegian.
Item translation	Forward backwards translation overseen by the project group.
Forward translators mother tongue is in the target language	Forward translators with Norwegian mother tongue.
Forward translators have expertise in the constructs	All forward translators and project group members had expertise with health care and patient engagement.
Backward translators mother tongue is in the original language	Backward translators with English language mother tongue.
Backward translators unfamiliar with the constructs measured	Backward translators were new to the field of patient engagement.
Translators work independently from each other	Communication with project group, not each other.
Clear description of how differences between the original and translated versions were resolved	Some words and response options were translated slightly differently and easily resolved and reviewed by the project group. Five new items relevant to Norway added.
Translation reviewed by committee (including original developers)	Project group reviewed the process and results and original developers were informed and accepted the methodology.
Report the translation process	Yes.
Cognitive interviews to assess comprehensibility, relevance, comprehensiveness: instructions, items, response options	Interviews with 12 patient representatives from 12 different institutions about relevant concepts to measure and mapping these onto the PPEET. 5 further items formulated and included. Translation with 5 items tested through interviews with 13 patient representatives, reviewed, and adjusted, until saturation.
Perform pilot study in population representing the target population	Survey of 47 patient representatives.

In accordance with guidelines for translation [11–15]

### Translation of the PPEET

The translation process followed methods recommended by COSMIN study design checklist for patient-reported questionnaires [11, 12], supervised by the project group comprising six health care experts (two nurses, an occupational therapist, two physical therapists, and a patient representative). Three independent, forward translations were undertaken by native Norwegian speakers. They were all familiar with the themes, health care system, patient engagement, and were fluent in the English language.

The three forward translations were combined into one version by the translation coordinator. In case of discrepancies, the three translators and the translation coordinator discussed and compared the results before coming to agreement. The final translated version was back translated by three native English translators working independently. The back translations were combined into one version by the translation coordinator. Discrepancies in the wording between the two translations were solved by discussion in the project group. Following comparisons of the back translation and the original version by the translation coordinator and project group, changes in wording were agreed upon. The project group then reviewed the final version and made a few final adjustments to improve readability of the translated text without compromising the content. The final translated version was used in the cognitive interviews.

### Interviews to assess content validity

The content validity of the PPEET was assessed in accordance with the COSMIN methodology for evaluating content validity [12]. Telephone interviews were conducted with PAB representatives designed to ascertain relevant concepts for patient engagement, which were then compared to the PPEET domains and items [14]. PAB representatives from 17 different institutions predominantly treating people with rheumatic and musculoskeletal diseases were invited by means of email. The sample size was determined by the point of saturation when no additional themes emerged. The interviews were conducted by one researcher to assess relevant concepts to measure from their perspective and followed a standardized semi structured interview guide with open questions developed by the project group specifically for this study. Concepts mentioned by the participants were thematically mapped onto the concepts of the PPEET by the researcher and discussed within the project group.

### Cogntive interviews

Telephone interviews were conducted with 13 patient representatives recruited from three institutions. Recruitment stopped when saturation was reached in terms of no additional information arising from subsequent interviews. The interviews were conducted by one interviewer and were designed to assess the relevance of the PPEET items for Norwegian health care, including comprehensiveness, as well as comprehensibility of all aspects of the questionnaire [15]. They followed a standardized interview guide based on the content of the PPEET, with open questions developed for the study to ascertain relevance. Results were discussed with three patient representatives from the project group.

### Data quality

The final survey was administered electronically through an online questionnaire to 150 PAB representatives registered in the overarching VIRKE and UNICARE rehabilitation organisations. Data quality including missing data, and response distributions including floor and ceiling effects were assessed. For the PPEET to be considered appropriate for use in Norwegian settings, missing data should be of a low level and there should be adequate use of response categories without large floor and ceiling effects.

The sample size was not large enough for extensive testing for measurement properties that follow international recommendations [10, 14]. However, responses to the background questions allowed hypothesis testing for validity [13] through comparisons with all items comprising the PPEET domains. Based on sample sizes, responses to background questions were collapsed into two meaningful categories. First, it was hypothesized that respondents reporting a middle or high level of awareness of their organisation’s approach to patient engagement, will have higher scores than those reporting less awareness. Second, it was hypothesized that respondents reporting fairly or very frequent collaboration with employees leading or supporting patient involvement, have higher scores than those reporting less frequent collaboration. Third, it was hypothesized that respondents reporting fairly to very frequent interaction with patient representatives would have higher scores than those respondents reporting less interaction. Fourth, it was hypothesized that those reporting established patient engagement in their organisation’s activities will have higher scores across PPEET items compared to those reporting less established patient engagement. The Mann-Whitney U test used to compare each of the resulting two groups in relation to the ordinal item data. Following existing recommendations, 75% of the results should be in accordance with the hypotheses [14, 15]. The Mann-Whitney U test used to compare each of the resulting two groups in relation to the ordinal PPEET item data. Following existing recommendations, 75% of the results should be in accordance with the hypotheses [13, 16].

### Ethics

Data protection procedures were followed and the protocol was approved by the local data protection officer (DS 00040). The study was not considered to fall under the medical research involving humans act and was conducted according to approved guidelines and the Declaration of Helsinki. All survey participants signed an informed consent form, and data were collected anonymously.

## Results

### PPEET translation

The forward translations showed no major content differences, but included a few minor variations in wording, vocabulary, and sentence structure. Examples of the slight discrepancies discussed included “the organisation” versus “your organisation”, “I can think of situations” versus “I know of situations”. These differences were discussed and resolved by the project group, and consensus was reached (Table 2). Backward translations clarified these wording discrepancies including “Leaders in the organisation show engagement” that could not be directly back-translated as “Organisational leaders demonstrate their commitment”, but the basic content was understood in both languages. Additionally, there were differences in the use of the simple present and present perfect for several items. Moreover, complex sentence structures from the translated version including question 2 about level of awareness (Table 3) «Hvordan vil du rangere kunnskapsnivået ditt? » was changed to «Hvor mye kunnskap har du? ». These differences were resolved by discussion. The project group then reviewed the final version and made a few final adjustments to improve readibility of the translated text without compromising the content.

**Table 2:** Specific discussion items

Item/term	English	Norwegian	Comment
28, 29 Appropriate	Overall, I believe this organisation devotes an **appropriate** level of resources to support its engagement initiatives.	Samlet sett tror jeg denne organisasjonen bruker **tilstrekkelig** med ressurser for å støtte tiltak for brukermedvirkning.	The translators suggested using “passelig” but was considered difficult to understand by patient representatives. “Tilstrekkelig” was preferred.
23 I can think of instances	**I can think of instances** where the input generated from PPE initiatives has influenced relevant governance decisions (i.e., at the Board level).	**Jeg er kjent med tilfeller** der innspill fra brukerutvalg har påvirket beslutninger innen denne organisasjonen.	The translators suggested” Jeg kan tenke på tilfeller” which is unclear.” Jeg er kjent med tilfeller” was preferred.
18, 20, 21 Contributions	Reports summarizing the **contributions** from PPE initiatives are shared with engagement participants and key stakeholders.	Rapporter som oppsummerer **innspill** fra brukermedvirkning deles med brukere, brukerutvalget og andre nøkkelpersoner.	The translators suggested “bidrag”, which with several meanings could be misunderstood. “Innspill” was preferred.
25, 26 Public	As a result of our PPE work, the organisation has identified shared goals with other groups (e.g., **public**, funders, community organisations, government departments).	Som et resultat av vårt arbeid med brukermedvirkning har organisasjonen identifisert felles mål med andre grupper (f.eks. **Befolkningen**, finansieringskilder, kommunale organisasjoner, departementer).	” Publikum” was suggested by the translators, but can have a slightly different meaning in Norwegian.” Befolkningen” was chosen.
12 A Committment to	**A commitment to** PPE values and principles is demonstrated through the structure of the organisation (e.g., dedicated PPE leadership positions).	**Verdier og prinsipper** om brukermedvirkning er definert i viktige organisasjonsdokumenter (f.eks. Oppdrag og visjon, strategi osv.).	” En forpliktelse til” was considered redundant in Norwegian. The project group chose starting the statement with “verdier og prinsipper”.

Public and Patient Engagement (PPE)

**Table 3:** Patient Advisory Board (PAB) respondent (*n* = 47) characteristics to the electronic survey

	Respondents	
	n	%
Female	29	62
Age, years		
30–39	1	2
40–49	10	21
50–59	11	23
60–69	13	28
70–79	11	23
80	1	2
Education		
Basic (≤10 yrs)	5	11
Secondary (11–13 yrs)	10	21
Degree	32	68
Years of experience		
< 2	9	19
> 2	38	81
Health region		
North	4	9
Middle	2	4
West	10	21
South-East	31	66
1 Organisational role^a^		
Board member	3	7
Member of the PAB	36	86
Employee	1	2
Manager	2	5
2 Level of awareness to approach for patient participation ^a^		
None	0	0
Low	0	0
Neither low or high	7	15
Medium	26	57
High	13	28
3 Frequency of interaction with patient representatives in organisation ^a^		
Not at all	2	4
Infrequently	10	22
Sometimes	19	41
Fairly frequently	14	30
Very frequently	1	2
4 Organisational stage of patient ^a^ participation		
Not started	1	2
Just beginning	11	24
Established/making progress	21	46
Well established	13	28

^a^ Questions from the The Public and Patient Engagement Evaluation Tool

### Interviews to assess content validity

Of the 17 invited, 12 (71%) PAB representatives from 12 institutions agreed to participate. The mean age was 53.8 (20 to 69) years and seven were female. In addition to the concepts arising that could be mapped onto the PPEET, information from interviews contributed to five new items relevant for the Norwegian version of the PPEET and were included in appropriate domains. These were: first, how often do you collaborate with employees that have responsibility for patient participation (*Background Questions*); second, are the responsibilities of the patient members clearly described (*Participatory Culture*); third, are the patient representatives treated equally alongside employees in joint meetings (*Influence and Impact*); fourth, do the patient representatives have voting rights in meetings with employees (*Influence and Impact*); and, fifth, overall I believe that patients, employees and the organisation are strengthened through patient involvement (*Final Thoughts*).

### Cognitive interviews

All 13 invited patient representatives took part in the interviews. Their mean age was 53.8 (20–69) years, eight were female and they had 1–35 years of experience with patient participation. Data saturation was reached following 10 interviews, the final three not contributing new information. Comments mainly related to suggestions for the use of different synonyms (i.e. recommendations and guidelines), layout and minor differences in semantic structure. The participants considered the content of the questionnaire highly relevant. In general, the change in PPEET response options throughout the questionnaire was considered a potential challenge for some respondents. However, because this was not identified as a problem by the developers or other studies, the scaling was not changed.

The project group reviewed the final version and made some adjustments to any ambiguities or deficiencies in clarity of the text.

### Data quality

Of the 150 invited to take part in the online survey, 47 (31%) completed a questionnaire. Table 3 shows their background characteristics along with responses to PPEET Background Questions. Based on the age categories, their mean age was approximately 60.5 (30 to 81) years and 29 (62%) were female.

PPEET items could be skipped within the on-line questionnaire, but there was no missing data. Table 4 shows that 12 items had responses to the “Don’t know” response category above 20%, including items relating to resources, job descriptions, training/materials to support staff, patient voting rights in meetings, instances where patient representatives input had influence, shared goals, and collaboration with other groups. By far the highest level of such responses at almost 50%, related to the item “Instances where patient representatives input influenced management decisions”.

**Table 4:** Descriptives for the The Public and Patient Engagement Evaluation Tool (PPEET) items (*n* = 47)

Domain/item ^a^	Mean (SD)	Floor %	Ceiling %	Don’t know %
*Policies and practices* ^b^				
6 Explicit strategy for PPE	3.87 (0.78)	0	15	2
7 Explicit strategies for recruiting participants	3.72 (1.00)	2	23	2
8 Clearly identified resources for PPE	3.62 (1.05)	2	15	28
9 Adequate PPE resources	3.32 (1.00)	0	9	21
10 Prepared reports of PPE	3.44 (1.05)	2	15	9
*Participatory culture* ^b^				
12 Commitment to PPE in key organisational documents	3.98 (0.83)	2	21	9
13 Commitment to PPE through organisational structure	3.52 (1.09)	4	17	6
15 Clear responsibilities for user representatives	3.67 (0.93)	2	13	4
16 Responsibilities in job descriptions of relevant staff	3.45 (0.91)	2	4	38
17 Comprehensive PPE training/materials to support staff	3.09 (0.95)	2	2	26
18 Adequate PPE training to support role	3.57 (0.99)	2	17	0
19 Leaders show commitment to using PPE input	3.91 (0.94)	2	28	2
20 Reports of contribution from PPE shared with participants	3.50 (0.93)	0	15	15
*Influence and impact* ^c^				
22 PPE contributions to organisation are identifiable	3.18 (0.60)	0	23	17
23 Leaders use input from PPE	3.24 (0.63)	0	28	19
24 User representatives equal with employees in meetings	3.41 (0.97)	9	62	6
25 User representatives voting rights in meetings with employees	2.90 (1.21)	15	28	36
26 Instances where PPE input had influence	2.94 (0.59)	2	9	26
27 Instances where PPE input influenced management decisions	2.83 (0.64)	2	4	49
*Collaboration and common purpose* ^c^				
29 PPE led to identifying shared goals with other organisations	3.52 (0.72)	0	6	34
30 PPE led to collaboration with other groups	3.44 (0.79)	0	2	28
*Final thoughts* ^b^				
32 Overall, the organisation has an appropriate PPE level	3.43 (1.08)	6	13	n/a
33 Overall, the organisation uses enough resources for PPE	3.28 (1.12)	4	13	n/a
34 Overall, patients, employees and the organisation benefit from PPE	4.15 (0.72)	0	32	n/a

^a^ 11, 14, 21, 28, 31 and 35 are questions with free text fields

^b^ Items 6–10, 12–20, 32–34 scored 1–5: strongly disagree, disagree, neither agree nor disagree, agree, strongly agree

^c^ Items 22–27, 29, 30 scored 1–4: never, seldom, some of the time, all of the time

Public and Patient Engagement (PPE)

Floor effects representing the worst possible levels of engagement ranged from 0% (for 8 items) to 15% for the item “Patient representatives voting rights in meetings with employees”. The latter was the only item where more than 10% scored at the floor. Ceiling effects, representing the best possible levels of engagement, were considerably higher and ranged from 2 to 62%, the latter relating to the item “Patient representatives equal with employees in meetings”.

The five sets of domain items were almost all approximately normally distributed with mean scores skewed towards the best possible levels of engagement. Item means on the five-point scale for the domains of *Policies and Practice*, *Participatory Culture* and *Final Thoughts* ranged from 3.09 to 4.15, for “comprehensive training/materials to support staff” and “overall, patients, employees and the organisation benefit respectively”. Item means on the four-point scale for the domains of *Influence and Impact*, and *Collaboration and Common Purpose* ranged from 2.83 to 3.52, for “Instances influenced management decisions” and “Patient representatives input led to identifying shared goals with other organisations” respectively. For the domains of *Policies and Practice* and *Participatory Culture*, modal values were found for the response categories of “neither agree nor disagree” or “agree”. For the domain of *Influence and Impact*, modal values were found for the response category of “sometimes” for all but the two items relating to equality and voting rights, which were more skewed with a modal value for the “all of the time” category. For *Collaboration and Common Purpose*, modal values were found for the “neither agree or disagree” and “agree” response categories for “Patient representatives input led to identifying shared goals” and “Patient representatives input led to collaboration” respectively. For the domain of Final Thoughts, all modal values were for the “agree” response category.

Table 5 shows the results of validity testing for PPEET items in relation to the background questions included in the questionnaire. In general, mean scores were slightly higher across PPEET items for: first, middle to high levels of awareness of patient involvement compared to lower levels; second, higher frequencies of collaboration with patient representatives compared to lower frequencies; third, higher levels of interaction with patient representatives compared to lower levels; and fourth, established patient involvement compared to unestablished or just beginning. There were 95 score comparisons based on available data; 76 (80%) were as hypothesized, and 20 (21%) showed statistically significant differences between the two categories. All domains except *Participatory Culture* met the 75% criterion (74–91%). As denoted by the results that are underlined in Table 5, the number of comparisons meeting the hypotheses was lowest for responses to the background questions relating to the frequency of interaction with patient representatives (58%), and highest for frequency of collaboration with employees that lead or support patient involvement with patient representatives (92%).

**Table 5:** Mean (SD) The Public and Patient Engagement Evaluation Tool item scores^a^ by responses to background questions (*n* = 47)

	Level of awareness^b^		Frequency of collaboration^c^		Frequency of interaction^d^		Stage of user involvment^e^	
	None/some	Mid-high	None/some	Often	Never/some	Often	None/begun	Established
Domain/item	(*n* = 33)	(*n* = 13)	(*n* = 31)	(*n* = 15)	(*n* = 32)	(*n* = 14)	(*n* = 12)	(*n* = 34)
*Policies and Practice*								
6 Explicit strategy	3.75 (0.76)	4.08 (0.76)	3.70 (0.84)	4.13 (0.52)	3.77 (0.76)	4.00 (0.78)	3.09 (0.94)	4.09 (0.51)**
7 Strategy - recruitment	3.50 (0.88)	4.15 (1.14)*	3.40 (1.00)	4.27 (0.70)**	3.65 (0.95)	3.79 (1.12)	3.00 (1.10)	3.91 (0.87)*
8 Clear resources	3.68 (1.09)	3.45 (1.04)	3.25 (1.07)	4.15 (0.80)*	3.75 (0.97)	3.38 (1.19)	2.83 (0.98)	3.78 (1.01)
9 Adequate resources	3.29 (0.96)	3.33 (1.16)	3.13 (0.92)	3.62 (1.12)	3.40 (0.91)	3.09 (1.22)	3.00 (0.93)	3.39 (1.03)
10 Reports	3.28 (1.10)	3.85 (0.90)	3.33 (1.11)	3.67 (0.98)	3.25 (1.11)	3.86 (0.86)	3.10 (0.99)	3.56 (1.08)
*Participatory Culture*								
12 Documents	3.93 (0.75)	4.08 (1.04)	3.70 (0.87)	4.47 (0.52)**	3.96 (0.64)	4.00 (1.18)	3.40 (0.97)	4.16 (0.72)*
13 Structure	3.47 (1.07)	3.54 (1.13)	3.17 (1.10)	4.14 (0.66)**	3.55 (1.06)	3.36 (1.15)	3.36 (0.92)	3.53 (1.14)
15 Clear responsibilities	3.61 (0.84)	3.85 (1.14)	3.41 (0.94)	4.20 (0.68)**	3.67 (0.80)	3.71 (1.20)	3.20 (1.03)	3.82 (0.87)
16 Job descriptions	3.59 (0.71)	3.18 (1.17)	3.41 (0.94)	3.45 (0.93)	3.59 (0.78)	3.18 (1.08)	3.43 (0.79)	3.43 (0.98)
17 Training/materials	3.00 (0.91)	3.22 (1.09)	2.86 (0.94)	3.42 (0.90)	3.08 (0.93)	3.00 (1.05)	2.90 (0.74)	3.13 (1.04)
18 PPE training	3.42 (1.03)	3.85 (0.80)	3.32 (0.98)	4.00 (0.85)*	3.50 (0.02)	3.64 (0.93)	3.17 (1.12)	3.68 (0.91)
19 Leaders commited	3.97 (0.82)	3.85 (1.21)	3.87 (0.94)	4.07 (0.96)	4.00 (0.86)	3.79 (1.12)	3.36 (1.12)	4.12 (0.81)
20 Reports shared	3.46 (0.88)	3.55 (1.13)	3.52 (0.96)	3.43 (0.94)	3.59 (0.89)	3.25 (1.06)	3.38 (0.52)	3.52 (1.03)
*Influence and Impact*								
22 Organisation	3.00 (0.58)	3.54 (0.52)*	3.00 (0.59)	3.50 (0.52)*	3.08 (0.58)	3.36 (0.63)	3.00 (0.50)	3.24 (0.64)
23 Leaders use input	3.08 (0.63)	3.64 (0.51)*	3.04 (0.62)	3.62 (0.51)*	3.16 (0.69)	3.42 (0.52)	2.89 (0.78)	3.36 (0.56)
24 Equality	3.30 (1.09)	3.69 (0.63)	3.29 (1.05)	3.67 (0.82)	3.31 (1.07)	3.64 (0.75)	2.70 (1.16)	3.64 (0.82)*
25 Voting rights	2.95 (1.16)	2.75 (1.49)	2.89 (1.23)	2.91 (1.30)	3.00 (1.11)	2.70 (1.49)	2.17 (1.17)	3.09 (1.20)
26 PPE influence	2.82 (0.66)	3.17 (0.39)	2.86 (0.73)	3.08 (0.28)	2.90 (0.70)	3.00 (0.41)	2.67 (0.52)	3.00 (0.61)
27 Management	2.69 (0.75)	3.00 (0.47)	2.83 (0.84)	2.82 (0.41)	2.85 (0.80)	2.80 (0.42)	–	2.83 (0.65)
*Collaboration and Common Purpose*								
29 Shared goals	3.20 (0.52)	4.10 (0.74)	3.45 (0.76)	3.60 (0.70)	3.45 (0.61)	3.60 (0.97)	3.40 (0.55)	3.52 (0.77)
30 Collaboration	3.17 (0.78)	4.00 (0.47)	3.35 (0.88)	3.54 (0.66)	3.38 (0.87)	3.50 (0.67)	3.43 (0.79)	3.42 (0.81)
*Final thoughts*								
32 Appropriate level	3.40 (0.90)	3.46 (1.51)	3.19 (0.98)	3.87 (1.19)*	3.44 (0.88)	3.36 (1.50)	2.75 (1.14)	3.65 (0.98)*
33 Uses resources	3.21 (0.99)	3.46 (1.45)	3.03 (1.05)	3.80 (1.15)*	3.28 (0.92)	3.29 (1.51)	2.67 (0.99)	3.50 (1.11)*
34 Overall benefit	4.03 (0.73)	4.46 (0.66)	4.10 (0.79)	4.27 (0.59)	4.09 (0.78)	4.29 (0.61)	3.67 (0.78)	4.32 (0.64)*

^a^ The underlined results do not meet the hypotheses. Asterisks denote statistically significant differences for Mann Whitney U Test: *P < 0.05; **P < 0.01

^b^ How would you rate your level of awareness of the organisation’s overall approach to public and patient engagement: “completely unaware, low level of awareness, neither aware nor unaware, some level of awareness” versus “middle or a high level of awareness”

^c^ How often do you collaborate with employees that lead or support patient involvement: “not at all, infrequently, sometimes” versus “fairly or very frequently”

^d^ How often do you interact with patient representatives associated with the organisation: “not at all, infrequently, sometimes” versus “fairly or very frequently”

^e^ At what stage would you say your organisation is when it comes to routinely engaging the public and/or patients in its activities: “not at all, just beginning”. versus “established or well established”

^f^ 11, 14, 21, 28, 31 and 35 are questions with free text fields

Very few items failed to meet two or more of the hypotheses and only the item “Responsibilities related to patient representation are clearly articulated in the job descriptions of staff who are leading and supporting these activities”, had three of four results inconsistent with the hypotheses. Five items had inconsistent results for two hypotheses within the PPEET domains of *Policies and Practice*, *Participatory Culture* and *Influence and Impact*.

## Discussion

Translation of the PPEET followed international recommendations and hence, the Norwegian and the Canadian version of the PPEET can be regarded as semantically equivalent [17, 18]. To ensure that items were relevant, interviews to assess content were conducted with the target population. The interviews were designed to elicit concepts considered relevant for measurement in terms of content validity, which were then mapped onto the original PPEET items. This permitted the identification of five additional items (see Table 5) assessing relevant themes in a Norwegian context and necessary for adequate cultural adaption. Further interviews were then conducted with the preliminary Norwegian PPEET to assess comprehensiveness, and relevance of the items (face validity) within the original version.

The additional PPEET items were deemed important in assessing patient representatives’ engagement in Norwegian health care institutions. Hence, their inclusion contributes content validity alongside existing items including within the PPEET domains of *Participatory Culture*, and *Influence and Impact*. The content of four items all relate to the role of the representative and suggest that frequency of collaboration, clear responsibilities, equality in meetings and voting rights are, in addition to the original PPEET items, important for engagement and influence. The fifth item relates to the overall belief in the importance of patient engagement and complements items within the domain of *Final Thoughts* relating to levels of engagement and resources.

The role of the additional five items within the Norwegian PPEET, will be assessed in accordance with international recommendations [13] in a larger sample size. These items will be analysed separately when comparing results of the PPEET cross-culturally.

Given the lack of an alternative instrument and the rigorous approach to translation, the Norwegian version of PPEET, named *Evalueringsverktøy for Brukermedvirkning* (EBNOR), can be recommended for assessing patient engagement in advisory settings in Norway. However, further testing is recommended in larger samples of the target population for other important measurement properties and more comprehensive validity testing.

The sample size of 47 meant that it was only possible to assess data quality and limited aspects of validity in terms of hypothesis testing at the item level. There was no missing data which is encouraging, but several items had large numbers of “Don’t know” responses. This shows the importance of this response category, with some items not being applicable to respondents who may have a limited experience or understanding of specific aspects of patient involvement in the organisation, Moreover, PABs are a recent development and not yet fully integrated into some organisational structures.

In general, item level data were approximately normally distributed with modal responses away from the end-points. Low floor effects were found across items, with only the item relating to voting rights in meetings, having more than 10% of responses indicating the lowest level of engagement. Whilst only two items had ceiling effects indicating the highest level of engagement, there was a positive skew towards high level of engagement across items. However, just eight items had ceiling effects above 20%. By far the highest at 62%, was related to patient representatives having equality in meetings with employees. These findings are encouraging in this Norwegian context because they indicate levels of engagement at the highest possible level. However, for 38% of the respondents there might be scope for improvement in levels of engagement within their organisation.

In spite of the low sample size, it was possible to undertake limited hypothesis testing through comparisons of item responses with responses to four of the background questions within the EBNOR tool. Such testing has not been undertaken for the original PPEET. The results gave an indication that the EBNOR is measuring what is intended through the results of comparisons with variables that were expected to be associated with item scores. The criteria used for such comparisons were developed within the field of health status and patient reported outcomes measurement [13], but have been widely applied to other types of measurement within health care, including patient preferences for engagement in health care [19], patient experiences of health care [20], and clinical rating scales [11, 21]. The vast majority (80%) of the results were satisfactory and met current recommendations [13].

In larger samples, testing could be extended to assess the reliability of individual items by means of a test-retest design. To date, the PPEET results have been reported at the item level as descriptive statistics and hence, an understanding of the stability of item responses will lend confidence to the interpretation of findings. Testing could be further extended to consider whether items might be grouped in multi-item domains by means of methods from classical or modern psychometric theory [13]. Domain scores that are based on theoretically and empirically supported multi-item scores have higher levels of reliability and validity. This makes them better suited than single items in comparisons of levels of engagement between organisations and over time, for purposes of evaluation. Moreover, a fewer number of domain scores will ease the burden of interpretation associated with 30 items, further contributing to its role in measuring the level and quality of patient engagement in organisations. However, single items might still prove useful in identification of specific aspects of patient engagement that warrant attention or improvement within an organisation.

Larger sample sizes are necessary for testing for the existence of domains by factor analytic methods. However, until such testing has been undertaken, the simple summation of item scores within domains such as *Policies and Practice* and *Participatory Culture* is not recommended. Future studies might also extend the hypothesis testing for validity undertaken here, through the inclusion of additional variables known, or expected, to be associated with levels of engagement.

### Strengths and limitations

Forward-backwards translation of the EBNOR tool followed international recommendations which together with the methods of testing are reproducible in other languages and cultures. Moreover, the study followed a multidisciplinary collaboration with strong involvement of the target group at all stages of testing. One of the researchers involved in this study is a PPI working for the Norwegian Rheumatism Association, which facilitates implementation. To date, research relating to the PPEET has included the development [8, 9] and reporting of results, including descriptive statistics at the item level [22]. The current study considered aspects of data quality including missing data and item distributions in addition to limited testing for validity based on hypotheses that followed widely used recommendations [13].

However, the sample size limited further testing for measurement properties including reliability and other aspects of validity including structural validity. The inclusion of additional variables that are known to be related to user engagement would have also provided further important evidence for the EBNOR tool. Future research should seek to assess the structural validity of the PPEET including evidence to support the proposed domains. Empirical support for these or other domains that are also supported by theory and expert opinion, can underpin the construction of multi-item scales with higher levels of validity and reliability than single items. It will be interesting to assess whether the findings are replicated across different health care systems and cultures. Future research should also assess the utility of the PPEET in a Norwegian context, including how the results of surveys can inform patient engagement initiatives.

The survey response rate of 31% was low and information on non-respondents was not available to permit a comparison of their background characteristics with those of respondents in order to assess response bias. It is possible that non-respondents felt that they lacked sufficient experience or knowledge of their organisation and hence were uncomfortable in providing their responses. It is also possible that they found the EBNOR items difficult to understand and were unable to complete the questionnaire in a manner that was acceptable to them. However, the results of testing for content validity and the cognitive interviews suggest otherwise, with the questionnaire proving acceptable and easy to understand. Future studies should seek to gain further background information from non-respondents which will allow testing for response bias. Moreover, non-respondents might be followed up and asked why they did not respond.

## Conclusion

The PPEET underwent a rigorous process of forward-backwards translation from English to Norwegian. Minor wording challenges were resolved through group discussions and the resulting EBNOR has adequate levels of comprehensibility and content validity. Five additional items were considered important in a Norwegian health care context, but for purposes of generalizability, these will not be included in cross cultural comparisons. Based on limited testing the EBNOR items have evidence for validity. Further testing for measurement properties is recommended. In the absence of an existing Norwegian instrument, the EBNOR should be considered for assessing patient engagement in Norwegian health care organisations.

## Data Availability

The data that support the findings of this study are available from the Diakonhjemmet Hospital research board but restrictions apply to the availability of these data, which were used under license for the current study, and so are not publicly available. Data is however available from the authors upon reasonable request and with permission of the research board.
